# Telemedicine in pre-hospital care: a review of telemedicine applications in the pre-hospital environment

**DOI:** 10.1186/s12245-014-0029-0

**Published:** 2014-07-05

**Authors:** Ahjoku Amadi-Obi, Peadar Gilligan, Niall Owens, Cathal O’Donnell

**Affiliations:** 1 Clinical Research Department, Royal College of Surgeons in Ireland, 123 St. Stephen’s Green, Dublin 2, Ireland; 2 Emergency Department, Beaumont Hospital, Beaumont, Dublin 9, Ireland; 3 School of Medicine, Royal College of Surgeons in Ireland, 123 St. Stephen’s Green, Dublin 2, Ireland; 4 National Ambulance Services, Oak House, Millennium Park, Naas, County Kildare, Ireland

**Keywords:** Telemedicine, Telecare, Telehealth, Teletrauma, Telestroke, eHealth, Mobile health, Pre-hospital care, Emergency medical services, Emergency medicine

## Abstract

The right person in the right place and at the right time is not always possible; telemedicine offers the potential to give audio and visual access to the appropriate clinician for patients. Advances in information and communication technology (ICT) in the area of video-to-video communication have led to growth in telemedicine applications in recent years. For these advances to be properly integrated into healthcare delivery, a regulatory framework, supported by definitive high-quality research, should be developed. Telemedicine is well suited to extending the reach of specialist services particularly in the pre-hospital care of acute emergencies where treatment delays may affect clinical outcome. The exponential growth in research and development in telemedicine has led to improvements in clinical outcomes in emergency medical care. This review is part of the *LiveCity* project to examine the history and existing applications of telemedicine in the pre-hospital environment. A search of electronic databases including Medline, Excerpta Medica Database (EMBASE), Cochrane, and Cumulative Index to Nursing and Allied Health Literature (CINAHL) for relevant papers was performed. All studies addressing the use of telemedicine in emergency medical or pre-hospital care setting were included. Out of a total of 1,279 articles reviewed, 39 met the inclusion criteria and were critically analysed. A majority of the studies were on stroke management. The studies suggested that overall, telemedicine had a positive impact on emergency medical care. It improved the pre-hospital diagnosis of stroke and myocardial infarction and enhanced the supervision of delivery of tissue thromboplasminogen activator in acute ischaemic stroke. Telemedicine presents an opportunity to enhance patient management. There are as yet few definitive studies that have demonstrated whether it had an effect on clinical outcome.

## Review

### Introduction

There is a critical global shortage of healthcare professionals. As a consequence, qualified professionals may not be physically present particularly in under-resourced regions, and providing quality healthcare may be quite challenging. This challenge can be tackled by providing specialist medical services using information and communication technologies to remotely located healthcare workers and patients where such expertise is not immediately available. This is known as telemedicine. In telemedicine, the client is separated from the expert in space [[1]]. The concept of telemedicine has been used in one form or another for centuries. Smoke signals were used in ancient African villages to alert adjoining villages of disease outbreaks, and bonfires were used to warn of bubonic plague in the Middle Ages in Europe. With advances in telecommunication, newer systems such as the telegraph were used to transmit medical information about epidemics and war casualties. However, the use of telemedicine was facilitated by the invention of the telephone in the nineteenth century. This culminated in one of the earliest recorded uses of information and communication technology (ICT) in telemedicine, when Einthoven, on 7th February 1906, transmitted electrocardiogram (ECG) tracings over telephone lines [[2]]. By the 1930s, medical information was being transmitted from remote regions of Australia and Alaska to specialist medical centres. With the invention of the television in the 1950s, advances in closed-circuit television and video conferencing led to the adoption of telemedicine in patient monitoring and consultations [[3]]. Perhaps, the earliest implementation of modern telemedicine was by the National Aeronautics and Space Administration (NASA) in the 1960s when it was used for remote physiological monitoring of astronauts during manned space flights [[4]]. NASA continued to play a pivotal role in the development of telemedicine with the development of the Space Technology Applied to Rural Papago Advanced Health Care (STARPAHC) project on the Papago Indian Reservation in Arizona, USA, in 1972. The STARPAHC project included a van equipped with an X-ray machine and other medical instruments, and it was staffed by two paramedics. The van was linked by two-way microwave transmission to the Public Health Service Hospital complemented by a remotely located clinic staffed by a physician assistant linked to the control centre in the hospital [[5]]. After the December 1988 earthquake disaster in Armenia, NASA established the first international telemedicine project known as the Spacebridge to Armenia that allowed telemedicine consultation between medical centres in the United States and Armenia [[6]]. By the 1970s, the National Library of Medicine funded research into the reliability of telemedicine via satellite communication to 26 sites in Alaska, USA. Improvements in telecommunications technology have led to advances in network infrastructure that enabled the development of high-definition live interactive video-to-video networks such as the European Union-funded *LiveCity* project. These, in turn, have led to increased telemedicine use over the past 40 to 50 years with a subsequent increase in research since the 1990s.

Telemedicine potentially holds great promise in facilitating emergency medical practice. It is increasingly being used in emergency medicine with an associated increase in published research. It is particularly suited to medical emergencies where treatment delays adversely affect clinical outcome. A typical scenario is ST elevated myocardial infarction (STEMI) where recognition of ECG changes by paramedics could facilitate early intervention and improve clinical outcome. However, recognition of ECG changes of STEMI by paramedics appears to be suboptimal [[7]], and adverse clinical events that occur during pre-hospital transportation [[8]] may also benefit from real-time clinician advice. Paramedics in ambulances have used telemedicine links with specialists to facilitate pre-hospital diagnosis and reduce treatment delays in stroke, myocardial infarction, and trauma. Telemedicine has also been used by emergency medicine doctors to supervise remotely located nurse practitioners and general practitioners in minor injury clinics [[9]].

This literature review is part of the emergency use case of the *LiveCity* project and analyses published studies to highlight the use of telemedicine in pre-hospital care.

### Methods

We performed an automated electronic search using the MeSH terms identified in Medline. The terms included the following: Telemedicine, Telecare, Telehealth, Teletrauma, Telestroke, eHealth, mobile Health, Pre-hospital care, and emergency care. The search terms were used as keywords on Medline, Excerpta Medica Database (EMBASE), Cochrane Database of Systemic Reviews (CDSR), Cochrane, and Cumulative Index to Nursing and Allied Health Literature (CINAHL). The search was then finalized using Boolean operators to combine (‘OR’) and cross-reference (‘AND’) between domains. The first ten pages of a basic web search using the Google search engine were analysed for relevant articles. A manual search was done by checking reference lists of selected articles and researching key authors.

Abstracts were independently reviewed by two of the authors, and the full text of articles that met the inclusion criteria were retrieved for further analysis. Included studies were collated and critically analysed based on their methodology and sample size to summarize their results.

Studies carried out between 1970 and 2014 that addressed telemedicine use in the emergency care of trauma, myocardial infarction, and stroke and studies whose outcomes included cost-effectiveness, feasibility, and clinical outcome were included in the review. Case reports and studies that were not published in English and did not address an aspect of emergency medicine were excluded from this review.

### Results

The initial literature search yielded 1,279 studies. Based on the inclusion criteria, 1,240 were excluded, while 39 were selected for further analysis. Twenty-five of the studies focused on stroke management, while five and nine were on myocardial infarction and trauma, respectively.

We found eight articles that were feasibility studies, while six articles explored the reliability of telemedicine. Four articles addressed the diagnostic accuracy of telemedicine, and three articles explored the use of telemedicine to reduce treatment delays. Thirteen studies used the ‘Hub and Spoke’ model, while five of the studies used a link between an ambulance and a hospital. Twenty-one of the studies reviewed did not clearly define the model used.

Table 1 shows the characteristics of the studies included. We found 2 randomized controlled studies, 10 case-control studies, and 27 observational and descriptive studies (1). The network types used in the studies were the following: mobile broadband in 8 and wired broadband in 31 of the studies included. The methodologies and subjects of the studies reflect the nascent nature of research in this field. Early studies address the feasibility, accuracy, and reliability of telemedicine use in clinical settings which have not been fully addressed due to design flaws identified in Tables 2, 3, and 4.

**Table 1:** Characteristics of the studies

	**Characteristic**	**Number of cases**
Study question	Accuracy	4
	Feasibility	8
	Treatment delay	3
	Clinical outcome	5
	Cost-effectiveness	1
	Reliability	6
	Others	12
Model of telemedicine	Hub-and-spoke	13
	Ambulance to hospital	5
	Others/unspecified	21
Technology	Computer based	35
	Smartphone based	4
Study type	Randomized control study	2
	Case-control	10
	Observational/descriptive	27
Network type	Mobile broadband (GSM)	8
	Wired broadband	31

**Table 2:** Stroke

**Authors**	**Journal/title**	**Methodology**	**Summary of findings**	**Comment**
Waite et al. [[10]]	*Journal of Telemedicine and Telecare* 2006;12:141–145. Telestroke: a multi-site, emergency-based telemedicine service in Ontario	Multicentre observational study to test the feasibility of telestroke consulting over a wired broadband. Neurologist in an academic centre carried the teleconsult for two peripheral emergency departments	88 consults with 24 patients receiving t-PA. Demonstrating that telestroke consulting was feasible	This is an observational study that demonstrates the feasibility of telemedicine across a network of hospitals
Hess et al. [[11]]	*Stroke* 2005;36:2018–2020. REACH: clinical feasibility of a rural telestroke network	Descriptive study of a new telestroke web-based consult linking eight rural hospitals with a neurology unit	194 stroke consults seen with 36 receiving tPA. Onset to treatment time dropped by 32 min during the course of the study	This is an observational study that demonstrates the feasibility of a hub and spoke telestroke system
Liman et al. [[12]]	*Stroke* 2012;43:2086–2090. Telestroke ambulances in prehospital stroke management. Concept and pilot feasibility study	To test the technical feasibility of telestroke over a 3G public network to a telemedicine-equipped ambulance with a simulated stroke patient	18 out of 30 scenarios could not be completed due to poor audiovisual quality. Poor network reliability was identified as a cause of unreliable results	This study highlights the challenges of a mobile telehealth platform using GSM network and demonstrates that it was not technically feasible at least in the location studied
Gonzalez et al. [[13]]	*Stroke* 2011;42:1522–1527. Reliability of prehospital real-time cellular video phone in assessing the simplified National Institutes of Health Stroke Scale in patients with acute stroke	Test of reliability of simplified NIHSS scale done remotely (over a cellular videophone on a 3G network) by a physician assisted locally by an emergency medical technician compared with bedside examination by physician	480 paired comparisons were done. The authors concluded that assessment over videophone was as reliable as bedside and could be a timely method for remote patient assessment	This study analyses the feasibility of conducting NIHSS assessment remotely over a 3G network but does not simulate real-life situation as the participants were not mobile
Pedragosa et al. [[14]]	*Journal of Telemedicine and Telecare* 2009;15:260–263 Impact of a telemedicine system on a stroke care in a community hospital	Retrospective case control of quality of care before and after introduction of telemedicine services	198 patients were managed with telemedicine compared to 201 cases the year before its introduction. Quality of care improved after introduction of telemedicine with reduction in transfers to stroke centre increased review by neurology specialist	This study compares a telestroke programme with historical control before introduction of this service and requires further studies to confirm their conclusions
Demaerschalk et al. [[15]]	*Telemedicine Journal and E-health* 2012;18:230–237 Efficacy of telemedicine for stroke: pooled analysis (STRokE DOC)	Pooled analysis of two prospective randomized controlled studies comparing telephone with telemedicine neurological consultation for stroke	276 pooled patients were evaluated. Telemedicine patients had better outcome with increased tPA treatment and reduced post-tPA bleeding, although 90-day mortality was similar	Although the two studies were identically designed, pooled analysis presents the challenge that the characteristics of the two sampled group may not be identical
Nelson et al. [[16]]	*Neurology* 2011;77(17):1590–1598 The cost-effectiveness of telestroke in the treatment of acute ischemic stroke	Cost-effectiveness of telestroke was analysed using a decision analytic model constructed by the team	There are higher upfront cost for telemedicine, but over the lifetime, telestroke is cost-effective	This study focuses only on analysing the cost-effectiveness of telemedicine in acute ischaemic stroke and not on other types of stroke. Also, due to lack of published data, some of the conclusions were based on assumptions and estimates
Demaerschalk et al. [[17]]	*Stroke* 2012;43:3095–3097 CT interpretation in a telestroke network: agreement among a spoke radiologist, hub vascular neurologist, and hub neuroradiologist	Randomized double-blind study analysing CT interpretation agreement among spoke radiologist, stroke neurologist, and central radiology adjudication committee	54 patients were recruited for the study. No significant difference in agreement between telemedicine group and standard method	The patients were randomized, but bias may be introduced by how the choice of telestrokologist is chosen. Sample size of study is small
Wang et al. [[18]]	*Stroke* 2003;34:e188–e191 Remote evaluation of acute ischemic stroke: reliability of National Institutes of Health Stroke Scale via telestroke	Case-control study comparing bedside telemedicine-based NIHSS assessment in stroke patients	20 patients were recruited for the study. There was no significant difference between bedside and telemedicine-based NIHSS	Small sample size. Larger sample size required to validate the result. Participating physicians not randomized introduce the possibility of bias
LaMonte [[19]]	*Journal of Stroke and Cerebrovascular Diseases* 2004;13(4):148–154 Shortening time to stroke treatment using ambulance telemedicine: TeleBAT	Case-control study comparing the reliability of NIHSS assessment of stroke video images transmitted through telemedicine ambulance (TeleBAT) and TV/VCR	Validity testing indicates that there was no significant difference between TV/VCR and assessment of video transmitted over the telemedicine system	Title indicates study on shortening of time to treatment but study is on analysing the reliability of radiological images transmitted from an ambulance
Handschu et al. [[20]]	*Stroke* 2003;34:2842–2846 Telemedicine in emergency evaluation of acute stroke : interrater agreement in remote video examination with a novel multimedia system	Case-control study comparing bedside with real-time remote video-based NIHSS assessment of stroke patients	41 patients were recruited for this study. It demonstrated that remote video-based NIHSS assessment was both feasible and comparable to bedside assessment	
Puetz et al. [[21]]	*Neurology* 2013;80:332–338 Reliability of brain CT evaluation by stroke neurologists in telemedicine	Retrospective analysis of the reliability and therapeutic impact of telemedicine-based CT interpretation in stroke patients	CT scans from 536 patients were analysed. There was high inter-observer agreement between telemedicine diagnosis and expert reviewers and minimal impact on clinical outcome	
Bergrath et al. [[22]]	*PLoS ONE* 2012;7(5):e36796 Feasibility of prehospital teleconsultation in acute stroke—a pilot study in clinical routine	A case-control study comparing telemedicine with standard paramedical care in the pre-hospital management of stroke	18 telemedical and 46 control patients were included in the study. No major effects on clinical processes but improvements in transfer of stroke specific data with corresponding clinical benefits	
Thomas et al. [[23]]	*Frontiers in Neurology* 2012;3:128 Variability in the perception of informed consent for IV-tPA	Retrospective analysis of the quality of informed consent taken during a telemedical consultation of stroke patients. Quality of 20 randomly selected video-taped consults was analysed by five raters	There was very high variability in the perception of consent, but 78.6% rated informed consent as adequate	Study would have been more informative if compared to face-to-face informed consent
Zaidi [[24]]	*Stroke* 2011;42:3291–3293 Telestroke-guided intravenous tissue-type plasminogen activator treatment achieves a similar clinical outcome as thrombolysis at a comprehensive stroke center	Prospective case-controlled study of telemedical vs. face-to-face management of stroke	Favourable outcome rates were similar between the two groups (42.1% versus 37.5%, *P* = 0.7)	No randomization. Face-to-face patients seen by hub team, while telemedical group seen my the spoke team
Chowdhury et al. [[25]]	*Postgraduate Medical Journal* 2012;88:134–137 Telemedicine versus face-to-face evaluation in the delivery of thrombolysis for acute ischaemic stroke: a single centre experience	Retrospective case-control study comparing telemedicine with face-to-face management of stroke patient	97 patients were assessed in the study; 52 (54%) face-to-face and 45 (46%) via telemedicine. Treatment delay was longer in the telemedicine group, but clinical outcome was similar	Method poorly described. Only CT scan appears to be viewed remotely. No information on whether patient assessment was done remotely. No info on how choice was made to use telemedicine
Pervez et al. [[26]]	*Stroke* 2010;41:e18–e24 Remote supervision of IV-tPA for acute ischemic stroke by telemedicine or telephone before transfer to a regional stroke center is feasible and safe	Retrospective case-control study comparing telemedicine with face-to-face supervision of IV-tPA in the management of stroke patient	296 patients were included in the study, of which 181 (61.1%) started IV-tPA remotely and 115 (38.9%) under direct supervision. The telestroke group had older patients on the average, but clinical outcomes were similar between both groups	
Meyer et al. [[27]]	*Journal of Stroke and Cerebrovascular Diseases* 2012;21(4):259–264 Assessment of long-term outcomes for the STRokE DOC telemedicine trial	Retrospective review of the 6-month outcome of telemedicine vs. telephone management	6-month outcome was not different between the two groups, and mortality was also the same at 18%	
Schwab et al. [[28]]	*Neurology* 2007;69:898–903 Long-term outcome after thrombolysis in telemedical stroke care	Prospective review of 3- and 6-month clinical outcomes after stroke thrombolysis with telemedicine supervision compared to face-to-face care in a stroke hub	11.2% mortality of the telemedical group compared to 11.5% in the face-to-face group in first 3 months. Favourable functional outcome was also similar between the two groups	Control group was treated in a stroke centre, while telemedicine group was treated in a community hospital
Audebert et al. [[29]]	*Stroke* 2006;37:1822–1827 Comparison of tissue plasminogen activator administration management between Telestroke Network hospitals and academic stroke centers: the Telemedical Pilot Project for Integrative Stroke Care in Bavaria/Germany	Prospective observational study comparing stroke thrombolysis in regional hospitals remotely supervised over a telemedicine link with thrombolysis in academic stroke unit	115 patients were treated in the regional hospitals, and 110 were treated in the stroke centres. The rate of IV-tPA was higher in stroke centres compared to regional hospitals although the quality of care was similar in both groups	Larger sample size is required to confirm the conclusions in this study
Ang et al. [[30]]	*European Journal of Emergency Medicine* 2013;20(5):322–326 Telestroke: rapid treatment of acute ischemic stroke patients using telemedicine in a Singapore emergency department	Retrospective observational analysis of the use of telemedicine in stroke management in a single centre (spoke). Teleconsultants were neurologist based at a specialist national centre (hub)	45 patients were enrolled into the telestroke programme, of which 18 were thrombolysed. Limited conclusion was reached due to the descriptive nature of the study	Limited conclusion can be drawn from this study due to the study design and small sample size
Switzer [[31]]	*Stroke* 2010;41:566–569 A telestroke network enhances recruitment into acute stroke clinical trials	Descriptive study analysing whether a hub and spoke telemedicine network enhances recruitment of patients for acute stroke trials	19 of 28 patients enrolled into two clinical trails were identified at the spoke level. Another nine patients were identified but could not be transported to the hub	This study explores an added advantage of telemedicine as an aid for patient recruitment into clinical studies
Agarwal et al. [[32]]	*Journal of the American Heart Association* 2014;3:e000408 Thrombolysis delivery by a regional telestroke network—experience from the UK National Health service	Observational study to demonstrate the safety and efficacy of out-of-hours telestroke service by a horizontal network of hospitals that have thrombolysis service during working hours. Out-of-hours service was provided by a rota of specialists across the network	A 4-month pilot phase with 15 patients demonstrated safety and feasibility. 164 patients were subsequently recruited over a 12-month period. There was significant increase in the number of thrombolysis carried out with outcomes that are comparable wit published studies	This study explores a different model to the traditional ‘hub and spoke’
Richard et al. [[33]]	*Neurological Science* 2014;35:683–685 Use of telemedicine to manage severe ischaemic strokes in a rural area with an elderly population	Observational study analysing the effectiveness and safety of a telestroke programme in a rural area with a high elderly population	53 patients were recruited to the study over a 16-month period. Outcome was worse than those in the published studies but the average age of this study group is much higher than those in other published data	Sample size is small, and study design does not allow clear conclusions from the study
Demaerschalk et al. [[34]]	*Stroke* 2012;43:3098–3101 Smartphone teleradiology application is successfully incorporated into a telestroke network environment	Case-control study assessing the reliability of smartphone-based CT interpretation by comparing it with PACS-based system	53 patients were recruited. There was an agreement (95% CI) between smartphone-based and PACS-based systems, suggesting that smartphone based systems are a reliable alternative	This study compares the interpretation by neurologists on smartphone with radiologists on PACS system, introducing a possible bias based on different specialties. Like-for-like comparison may be required to validate their study

**Table 3:** Myocardial infarction

**Authors**	**Journal/title**	**Methodology**	**Summary of findings**	**Comments**
Terkelsen et al. [[35]]	*European Heart Journal* 2005;26(8):770–777 Reduction of treatment delay in patients with ST-elevation myocardial infarction: impact of pre-hospital diagnosis and direct referral to primary percutaneous coronary intervention	Comparative analysis of treatment delay in patients diagnosed with ST-segment elevated myocardial infarction (STEMI) during the pre-hospital period or in hospital	Treatment delay was significantly reduced in patients diagnosed during the pre-hospital period	
Zanini et al. [[36]]	*Journal of Cardiovascular Medicine* 2008;9:570–575 Impact of prehospital diagnosis in the management of ST elevation myocardial infarction in the era of primary percutaneous coronary intervention: reduction of treatment delay and mortality	Observational study comparing STEMI patients transported with telemedicine-supported ambulance with patient diagnosed in hospital	399 patients were recruited: 136 via telemedicine, while 263 came directly to the hospital. There was significant reduction treatment delay in the telemedicine compared to the in-hospital group	This is an observational study. Randomized controlled study will be required to validate the results
Brunetti [[37]]	*European Journal of Cardiovascular Prevention & Rehabilitation* 2010;17:615 Telecardiology improves quality of diagnosis and reduces delay to treatment in elderly patients with acute myocardial infarction and atypical presentation	Observational study to analyse the effectiveness of telemedicine in the diagnosis of STEMI	Of the 27,841 patients recruited for the study, 534 had ECG changes consistent with STEMI. Telemedicine improved the quality of diagnosis of STEMI and also led to reduction in treatment delay	Large prospective study with statistically significant conclusions
Terkelsen et al. [[38]]	*Journal of Internal Medicine* 2002;252:412–420 Telemedicine used for remote prehospital diagnosing in patients suspected of acute myocardial infarction	Observational study analysing the technical feasibility of diagnosis of myocardial infarction from ECG transmitted from an ambulance over a GSM network	Of the 250 patients with ECG transmitted, 214 (86%) were technically successful. Telemedicine also reduced treatment delays	This study compared telemedicine-equipped ambulance with regular ambulance. No selection mechanism was used to decide which ambulance transport a patient
Sejersten et al [[39]]	*American Journal of Cardiology* 2007;11:038 Effect on treatment delay of prehospital teletransmission of 12-lead electrocardiogram to a cardiologist for immediate triage and direct referral of patients with ST-segment elevation acute myocardial infarction to primary percutaneous coronary intervention	Case-control study to determine whether treatment delays in myocardial infarction can be reduced by transmitting pre-hospital 12-lead ECG directly to cardiologist phone	Of the 243 patients enrolled in the study, 184 were referred for percutaneous coronary intervention (PCI). ECG transmission was successful in 94%. 72% of the telemedicine group underwent PCI within 90 min of 911 call compared to 13% in the historical controls	Historical controls were used for the study, indicating the possibility of bias

**Table 4:** Trauma

**Authors**	**Journal/title**	**Methodology**	**Summary of findings**	**Comment**
Duchesne et al. [[40]]	*The Journal of Trauma* 2008;64(1):92–97 Impact of telemedicine upon rural trauma care	Comparative analysis of the outcomes before and after the introduction of telemedicine in the trauma management in rural hospitals	Telemedicine improved trauma evaluation and management and led to reduction in hospital cost and mortality	The use of historical controls in this study introduces bias that compromises the conclusions of the study
Saffle et al. [[41]]	*The Journal of Trauma* 2009;67(2):358–365 Telemedicine evaluation of acute burns is accurate and cost-effective	Retrospective comparative analysis of burns evaluation before and after the introduction of telemedicine	80 patients were recruited to the telemedicine arm, while 28 were recruited during the same period pre-telemedicine. Burns assessment by telemedicine is both accurate and low cost	This study uses historical controls in its analysis. Its conclusion will require conformation by a large randomized control study
Boniface et al. [[42]]	*American Journal of Emergency Medicine* 2011;29:477–481 Tele-ultrasound and paramedics: real-time remote physician guidance of the Focused Assessment With Sonography for Trauma examination	Analysed whether paramedics could perform focused assessment with sonography for trauma (FAST) under remote guidance by an emergency physician	51 paramedics were able to complete FAST with 100% of the view under emergency physician guidance	This is an observational study that demonstrates the feasibility of telemedicine-guided FAST by novice paramedics. Further studies will be needed to explore its accuracy
Charash et al. [[43]]	*Journal of Trauma* 2011;71(1):49–54 Telemedicine to a moving ambulance improves outcome after trauma in simulated patients	Prospective double-blind study of simulated trauma patients. The study compares the outcomes of trauma care in a moving ambulance between telemedicine group and non-telemedicine control	Telemedicine to a moving ambulance improves care and successfully guide EMTs through needle thoracostomy and pericardiocentesis	This is a well-designed simulation study that will require investigation with real-life scenarios to confirm their findings
Rogers et al. [[44]]	*The Journal of Trauma Injury, Infection, and Critical Care* 2001;51:1037–1041 The use of telemedicine for real-time video consultation between trauma center and community hospital in a rural setting improves early trauma care: preliminary results	Observational study analysing whether real-time telemedicine consult with a trauma surgeon by community hospital emergency department positively affects care	26 teleconsults were carried out by trauma surgeons over an 8-month period, and survey indicated that 80% felt telemedicine improved patient care	This study is descriptive, and no conclusions were reached
Wallace et al. [[45]]	*Journal of Telemedicine and Telecare* 2007;13:282–287 A cohort study of acute plastic surgery trauma and burn referrals using telemedicine	Prospective cohort study comparing the management of patients referred to a burns unit with/without telemedicine (store-and-forward)	Telemedicine group was more likely to be booked directly to day surgery without the need for initial assessment. Of the 34 responders to the survey, 31 thought telemedicine improved patient management	The authors did not specify the method of selection of which facilities had telemedicine units installed. There is also very limited description of the facilities, making comparison very difficult
Tachakra et al. [[46]]	*Journal of Telemedicine and Telecare* 2011;17:350–357 A comparison of telemedicine with face-to-face consultations for trauma management	Case-control study comparing the diagnostic accuracy of telemedicine with face-to-face in minor trauma	200 patients were recruited for the study. There was a very high diagnostic accuracy both in the final diagnosis and in the clinical features	Physicians involved were not blinded or randomized
Rörtgen et al. [[47]]	*Resuscitation* 2013;84(1):85–92 Comparison of physician staffed emergency teams with paramedic teams assisted by telemedicine—a randomized, controlled simulation study	Randomized controlled study comparing emergency physician team with telemedicine-assisted paramedic teams in management of four simulated clinical scenario	Total of 31 scenarios were completed by both groups, and there was no statistical difference between the groups' performance	Well-designed study that demonstrates feasibility and quality in a simulation
Tachakra et al. [[48]]	*Journal of Telemedicine and Telecare* 2000;6:330–334 A follow-up study of remote trauma teleconsultations	Retrospective review of patients in a minor trauma telemedicine programme for diagnostic accuracy and sequelae of initial trauma	Diagnosis was wrong in 2% of patients that were managed with telemedicine. The results were similar with those of face-to-face	Observational study

### Discussion

There has been an exponential growth in the number of telemedicine articles published since the mid-1990s. This review noted the highest amount of research into telemedicine use in stroke care. Trauma and myocardial infarction have seen much less telemedicine-related research.

#### Stroke

Telemedicine in stroke management has undergone the most extensive study of all areas examined. Its use is feasible [[10],[11]] but dependent on the technical performance of the telemedicine equipment and broadband infrastructure [[12],[21]]. Due to its novel uses, medico-legal concerns have led to questions about the relevance and clarity of communication during informed consent. However, analysis of video-taped telemedicine consultations of acute stroke patients before intravenous administration of tissue plasminogen activator showed that 80% of observers rated informed consent as adequate [[23]]. Administration of tissue thromboplasminogen activator (tPA) within 3 to 4.5 h [[49]–[51]] of an acute ischemic stroke remains the gold standard in its management. However, this approach is restricted by time constraints and requires the supervision of a clinician with expertise in stroke management, and as a result, there is a disappointingly low utilization of thrombolysis in ischaemic stroke [[52],[53]]. Where available, integrating stroke specialists in pre-hospital stroke response teams significantly reduces time to treatment [[54]]. This is however not possible in a large proportion of locations where there is a limited availability of stroke specialists. Remote access to a stroke specialist is now possible, and recent studies comparing in-person consultation with remote consultation suggest that telemedicine is a promising solution to the lack of local expertise. The National Institute of Health Stroke Scale (NIHSS) assessment of stroke patients using telemedicine is as reliable as face-to-face assessment [[20]]. And radiological review of brain CT in stroke management is both feasible and reliable [[34]]. In the ‘hub and spoke model’, under served areas where stroke management expertise is lacking (i.e. spoke), telemedicine provides an ideal opportunity for supervision by a centrally located stroke expert (hub). Analysis of clinical outcomes of patients managed using this model suggest that although there is increased consultation, the quality of care remains similar and there was no statistical difference between telemedicine and face-to-face consultation, in short-term [[14],[15]] and long-term [[25],[26]] mortality. In the context of budgetary constraints, a cost-effectiveness analysis indicated that telemedicine is more expensive than usual care [[16]] partly due to high upfront equipment cost. However, there is the potential for significant cost savings due to reduced length of hospital stay [[55]].

#### Trauma

The effects of telemedicine on trauma management have not been as widely studied as in stroke, in the emergency medical services. Telemedicine has been deployed in major disasters such as the Armenian earthquake disaster in 1988. It is well suited to the management of major incidents where an acute deficit of healthcare professionals can be ameliorated by teleconsultation [[56]]. Where local expertise is lacking, teleradiology has improved diagnosis and reduced expensive transfer of trauma patients [[57]]. Analysis of the impact of telemedicine on emergency medical services suggests a reduction in mortality and hospital cost [[40]]. In a hub and spoke model of a central burns unit and three peripheral hospitals, telemedicine use led to increased consultation, but burns assessment was as accurate as face-to-face assessment and reduction in transfers to burns units led to significant cost savings [[41]]. Interestingly, paramedics that were guided by an emergency medicine clinician could obtain interpretable focused assessment with sonography for trauma (FAST) ultrasound [[42]], recognize key physical signs, and make better management decisions [[43]]. The use of a telemedicine referral in an acute burns unit led to a reduction in admission that could reduce hospital costs [[45]].

#### Myocardial infarction

The ideal recommendation for reperfusion of STEMI is within 2 h of first medical contact [[58]]. The requirement for urgent management of patients with myocardial infarction can be facilitated by the use of telemedicine for diagnosis and treatment. Efforts to shorten treatment delay are crucial, and various studies have been published addressing this challenge. Patient transfer directly to percutaneous coronary intervention (PCI) laboratory after pre-hospital diagnosis of STEMI in a telemedicine-equipped ambulance reduced treatment delay [[35],[36]] and reduced mortality from myocardial infarction [[59]]. To expedite reduction in treatment delay, accurate ECG diagnosis of STEMI remains crucial. Currently computer [[60]] and paramedics [[7]] ECG interpretation are not reliable enough to enhance patient triage for urgent PCI.

## Conclusion

This review found limited conclusive studies for the effectiveness of telemedicine in emergency medicine. The best evidence is in stroke management where conclusive evidence of the significant positive effect of telestroke on clinical outcome has led to its recommendation for stroke management. Telemedicine appears to have a significant impact on the quality of ECG interpretation, but there is as yet no conclusive evidence that telemedicine affects clinical outcome in myocardial infarction. We could find very few studies that critically analysed telemedicine use in the pre-hospital care of trauma. Studies have demonstrated that burns assessment using telemedicine was as accurate as face-to-face assessment.

The proliferation of smartphones, tablets, and other mobile electronic devices creates an opportunity to extend standard professional health care particularly in medical emergencies where urgent intervention could reduce mortality and improve quality of life. Telemedicine could enhance emergency medical services by helping expedite urgent patient transfer, improve remote consultation, and enhance supervision of paramedics and nurses.

However, in order to regulate and standardize practice, more research is required. Particular emphasis should be on better study design and larger sample size to improve the reliability of results and conclusions. A large proportion of the studies analysed focused on ambulance mounted equipment. Wearable technology such as head-mounted displays that will allow paramedics reach patients *in situ* may improve early pre-hospital diagnosis and should be investigated. To further reduce response times, consideration should also be given to incorporating smartphone technology into emergency systems and thus facilitate patient or bystander incident reporting. Although technological advances will continue to outpace their utilization in clinical practice, incorporating emerging technologies into medical practice holds promise in improving care and enhancing clinical outcomes, and researchers must continue to evaluate the effectiveness of telemedicine so that communication technology-assisted care is optimized.

## Competing interests

This work is funded by the European Union through the *LiveCity* project. The authors report no conflict of interest.

## Authors’ contributions

Conception and design of the review was done by AA, PG, and NO. Articles were independently reviewed by AA and NO. PG provided study oversight. All authors participated in the critical review and revision of manuscripts. All authors read and approved the final manuscript.
